# A Novel Joint Adversarial Domain Adaptation Method for Rotary Machine Fault Diagnosis under Different Working Conditions

**DOI:** 10.3390/s22229007

**Published:** 2022-11-21

**Authors:** Xiaoping Zhao, Fan Shao, Yonghong Zhang

**Affiliations:** 1 School of Computer and Software, Nanjing University of Information Science and Technology, Nanjing 210044, China; 2 College of Aerospace Engineering, Nanjing University of Aeronautics and Astronauties, Nanjing 210016, China; 3 School of Automation, Nanjing University of Information Science and Technology, Nanjing 210044, China

**Keywords:** transfer learning, rolling bearing, intelligent fault diagnosis, joint adversarial domain adaptation, convolutional neural network

## Abstract

In real-world applications of detecting faults, many factors—such as changes in working conditions, equipment wear, and environmental causes—can cause a significant mismatch between the source domain on which classifiers are trained and the target domain to which those classifiers are applied. As such, existing deep network algorithms perform poorly under different working conditions. To solve this problem, we propose a novel fault diagnosis method named Joint Adversarial Domain Adaptation (JADA) for fault detection under different working conditions. Our approach simultaneously aligns marginal distribution and conditional distribution across the source and target through a unified adversarial learning process. JADA aims to construct domain-invariant and category-discriminative feature representation that is effective and robust for substantial distribution difference caused by working conditions. We also introduce a supervision signal, namely center loss, that penalizes the distances between the deep features and their corresponding class centers. This makes the learned features better equipped with more discriminative structures and effectively prevents mode collapse. Twenty-four transfer fault diagnosis tasks based on two experimental platforms were conducted to evaluate the effectiveness of the proposed methods. Extensive experiments verified that the JADA can significantly outperform several popular methods under different transfer diagnosis tasks.

## 1. Introduction

Rolling bearings are widely used in manufacturing as an important part of rotating machinery, and their failure directly impacts the performance of the machinery [1]. Currently, the intelligent fault diagnosis could be driven by deep learning (DL) [2,3,4,5]. These approaches rely on a large amount of labeled data. However, it is expensive and time-consuming to accumulate many data. Factors such as equipment wear and degradation, changes in operating conditions, and external noise interference cause inevitable data distribution differences, making it expensive to mark the health status of the device corresponding to the data. Therefore, many efforts are needed to identify how to use the data with a known health status to identify the target data subject to different distributions as well as improve the accuracy of unsupervised health status recognition.

The present study was undertaken with the aim to use rich labeled data in relevant source domains to complete the identification of the health status of rolling bearings under unknown operating conditions without shutting down the equipment. Compared with the existing DL-based methods that depend on conditions such as the consistent distribution of source domain data (training data) and target domain data (the data of the actual deployment model) and a sufficient amount of labeled data, this fault diagnosis method is consistent with the actual engineering application scenarios. Our approach is more suitable and has the following characteristics: (1) the ability to use existing fault diagnosis knowledge to assist the learning of fault information under different working conditions; (2) the ability to suppress the impact of the difference in data distribution caused by factors such as variable working conditions and equipment wear to diagnose the model, and the performance is applicable to a wide range of scenarios; (3) pre-training on source domain data improves the diagnosis efficiency of the model, and the diagnosis results are more time-sensitive.

In recent years, the intelligent fault diagnosis method based on deep transfer learning has rapidly developed in order to deal with the problem of negligible or no data annotation in actual engineering scenarios [6,7,8]. The basic process of this method is to learn the information obtained from easily accessible fault data (source domain), and to help identify costly failures (target domains) in data annotation. To solve the problem of the availability of only a small amount of labeled data [9], Fine-Tune is the most commonly used method [10]. Many researchers [11,12,13] used relevant data to complete the pre-training of deep convolutional networks and using only a small amount of labeled data to fine-tune the last layers. However, there are bottlenecks in the diagnostic accuracy of the aforementioned methods, and fine-tuning cannot adapt to the influence of changes in data distribution in different fields, and cannot solve the problem of no labeled data availability. The difference in data distribution is the main obstacle for the intelligent fault diagnosis model to adapt to the target task. In order to alleviate its impact on the diagnosis performance, domain adaptation (DA) has been proposed as a cross-domain transfer learning method [14,15,16]. This implies learning a new feature space, establishing connection between the source and target domains, and reducing the difference between the domains; it is applied to the situation where the source domain label is available and the target domain label is not available. Lei et al. [17] combined the residual network with the maximum mean difference (MMD) term and pseudo-label learning, and proposed an unsupervised domain adaptive method. In addition, they also proposed an approach based on adversarial learning and MMD. Domain adaptive networks are used for knowledge transfer in different directions [18]. Wen et al. [19] realized the distribution matching of the source domain and target domain data by adding a DA layer in the autoencoder model. In addition, some researchers constructed an intelligent fault diagnosis model based on migration component analysis [20] and joint distributed adaptation [21]. However, the aforementioned domain-adaptive methods only matched the feature distributions of the source and target domains [22], ignoring the relationship between the sample categories in different domains.

With the aim to mitigate the aforementioned shortcomings, here, we propose a joint adversarial domain adaptation (JADA) fault diagnosis method to realize the intelligent fault diagnosis of rolling bearings under variable operating conditions. First, the labeled source domain data are used to perform supervised learning on the source domain feature extractor and classifier. Next, adversarial learning is employed to optimize the target domain feature extractor and simultaneously adapt the edge distribution and conditional distribution across domains. Finally, the accurate identification of the health status of the samples in the target domain is achieved. The experimental results showed that the JADA method is significantly advantageous for the learning of cross-domain diagnostic information, compared to the commonly used transfer learning methods. The rest of this paper is organized as follows. In Section 2, we begin by describing the domain adaptation tasks of this study. Section 3 details the proposed JADA model including three stages. Furthermore, its implementation details are presented. Section 4.1 conducts two domain adaptation cases and the corresponding analyses. The conclusions are drawn in Section 5.

## 2. Preliminaries

In this part, several related definitions for the mechanical fault diagnosis with DA and JADA techniques are introduced in detail.

Suppose that domain data are composed of data space X and a marginal probability distribution P(X), e.g., D={X,P(X)}, where X∈X. The task refers to the goal of fault diagnosis learning, which is defined as T={Y,f(X)}, where Y is the label space corresponding to the feature, and f(·) denotes the prediction function. In addition, f(X)=Q(Y∣X) is the conditional probability distribution, and Y∈Y. The main challenge of the unsupervised DA are summarized below.

(1) The labeled data only exist in the source domain, and there are no labeled data in the target domain. We denote the source domain as $D_{s}={\left\{\left(x_{i}^{s},y_{i}^{s}\right)\right\}}_{i=1}^{n_{s}}$, and the target domain as $D_{t}={\left\{x_{i}^{t}\right\}}_{i=1}^{n_{t}}$, where $n_{s}$ and $n_{t}$ indicate the number of source and target samples, respectively, $x_{i}$ represent the i-th data example, and $y_{i}$ is the corresponding category label for $x_{i}$.

(2) The source and target domains are different in both the marginal and conditional distributions, e.g., $P_{s}\left(X_{s}\right)\neq P_{t}\left(X_{t}\right),Q_{s}\left(Y_{s}|X_{s}\right)\neq Q_{t}\left(Y_{t}|X_{t}\right)$.

The objective of JADA is to obtain a feature extractor f(·) which can learn the domain-invariant and category-discriminative features, and then generate a target distribution that can maximize the performance of classifying the samples in $D_{t}$ without accessing its label, in the feature space.

## 3. The Proposed Method

**Figure 1:**
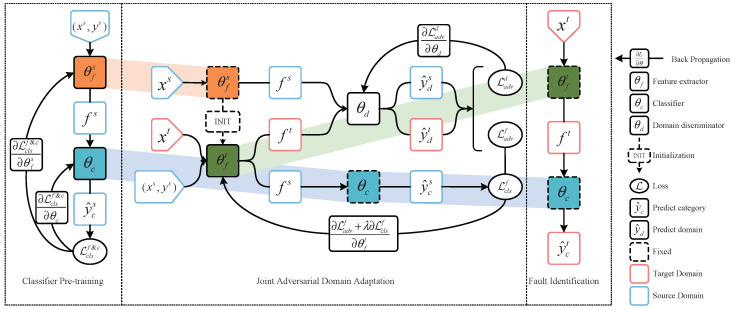
Fault diagnosis process of JADA.

In this paper, we propose the JADA method, which is an intelligent diagnosis approach that can capture global information as well as category-wise intrinsic information to enhance the distribution matching between the source and target domains. Generally, the proposed framework contains three stages: classifier pre-training, JADA, and fault identification, as displayed in Figure 1. The steps of each stage are introduced as elaborated below.

Classifier pre-training stage: Use the labeled data in the source domain to complete the supervised training of the source domain feature extractor and classifier. First, input the source domain samples into the feature extractor to obtain the feature representation of each sample; then, use the classifier to classify the sample features and calculate the cross-entropy loss of the classification result; finally, the feature extractor and classifier are continuously optimized through back propagation. The parameters enable the feature extractor to extract the effective features, and the classifier can accurately classify the extracted features.

JADA stage: Training the target domain feature extractor and domain discriminator through joint adversarial learning. First, alternately optimize the domain discriminator and feature extractor while improving the domain discrimination ability of the domain discriminator as well as the ability of the feature extractor to extract domain invariant features; simultaneously, use the source samples to optimize the category-wise distinction of the features extracted by the feature extractor; ultimately reduce the marginal distribution and conditional distribution difference between the source domain and the target domain.

Fault identification stage: Use the target feature extractor and classifier to diagnose faults in the target domain. First, fix the parameters of the target domain feature extractor and the classifier constructed in the two above stages; second, use the target feature extractor to extract the target sample to obtain the feature representation; finally, use the classifier to identify the feature of the sample fault type, and complete the diagnosis of the unsupervised cross-domain fault samples. The implementation details of the above stages are described in the following sections.

### 3.1. Classifier Pre-Training

**Figure 2:**
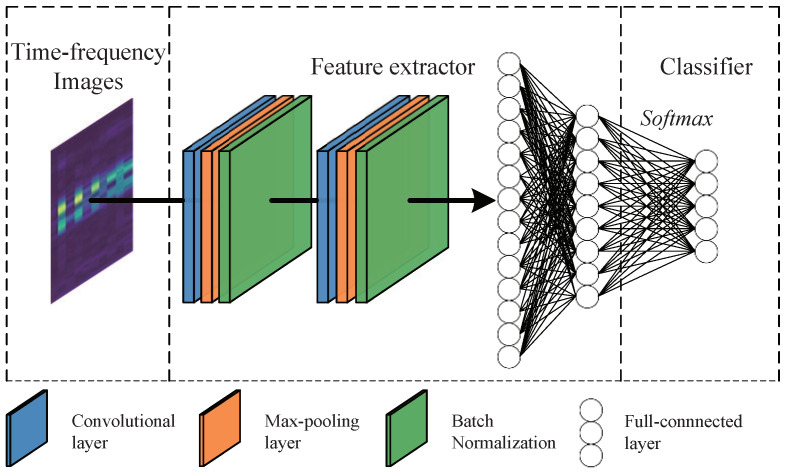
Feature extractor and classifier model.

The proposed method learns the domain invariant features of the source and target domains while minimizing the distribution distance of the features extracted from both these domains, such that only the source classifier can be directly applied to the target domain, eliminating the need to learn a separate target classifier, i.e., $\theta_{c}=\theta_{c}^{s}=\theta_{c}^{t}$. Therefore, we first complete the construction of the classifier in this stage. To effectively extract the features, a convolutional neural network (CNN) is designed as the feature extractor $\theta_{f}^{s}$, and the classifier module $\theta_{c}$ is composed of fully connected layers, as shown in Figure 2.

From the network structure shown in Figure 2 and the classifier pre-training stage illustrated in Figure 1, it can be seen that the feature extractor $\theta_{f}^{s}$ takes three-channel time–frequency images $x^{s}$ as the input, and the convolution is initially conducted to optimize the features. Then, a nonlinear activation function is added to enhance the fitting ability of the module, and batch normalization is performed to make the results of each convolutional layer conform to the standard normal distribution, eliminating the magnitude difference between the hidden layers; this can prevent the problem of gradient disappearance to a certain extent. Then, in the process of feature map down-sampling, max-pooling is performed to reduce the number of trained parameters while retaining more texture information. The fully connected layer adequately outputs the feature representation $f^{s}$ of the source samples, which is expressed as follows.

(1) $$f^{s}=\theta_{f}^{s}\left(x^{s}\right)$$

In terms of classification, the classifier $\theta_{c}$ is composed of fully connected layers, which take the features $f^{s}$ expressed in Equation (1) as the input, and the softmax function is used in the classifier to predict the labels ${\hat{y}}_{c}^{s}$ of the linear prediction result output by the fully connected layer, which is expressed as follows.

(2) $$\begin{array}{cc} {\hat{y}}_{c}^{s}=\underset{i}{\arg\max} & \left(\frac{\exp\left({\left(w_{i}^{\theta_{c}}\right)}^{T}f^{s}\;+\;b^{\theta_{c}}\right)}{\sum_{j=1}^{m}\exp\left({\left(w_{j}^{\theta_{c}}\right)}^{T}f^{s}\;+\;b^{\theta_{c}}\right)}\right),\\ & i=1,\ldots ,m \end{array}$$

where $w_{j}^{\theta_{c}}$ and $b^{\theta_{c}}$ represent the classifier weights and the classifier bias. To enhance the discriminative power of the extracted features and reduce intra-class variations, cross-entropy loss and center loss [23] are used to train the feature extractor and classifier for feature learning in a joint supervision method. The loss formulation is given in Equation (3).

(3) $$\begin{array}{cc} L_{\mathrm{cls}\;} & =L_{ce}+\kappa L_{C}\\ \; & =-\sum_{i=1}^{m}y_{i}^{s}\log{\hat{y}}_{i}^{s}+\frac{\kappa }{2}\sum_{i=1}^{m}{∥f_{i}^{s}-c_{y_{i}}∥}_{2}^{2} \end{array}$$

where $c_{y_{i}}$ denotes the $y_{i}$th class center of the features. To improve the computational efficiency and avoid large perturbations caused by a few mislabeled samples, we update the centers with respect to the mini-batch and use a scalar α to control the learning rate of the centers, which is expressed as follows:

(4) $$\begin{array}{cc} \Delta c_{j} & =\frac{\sum_{i=1}^{m}\delta \left(y_{i}=j\right)\cdot \left(c_{j}-x_{i}\right)}{1+\sum_{i=1}^{m}\delta \left(y_{i}=j\right)}\\ c_{j}^{t+1} & =c_{j}^{t}-\alpha \cdot \Delta c_{j}^{t} \end{array}$$

where $\delta \left(y_{i}=j\right)=1$ if the condition $y_{i}=j$ is satisfied, and $\delta \left(y_{i}=j\right)=0$ if not, and α is restricted in [0,1]. Moreover, the formulation introduces a scalar κ to balance the cross-entropy loss and center loss; when κ is taken as 0, the loss function $L_{\mathrm{cls}\;}$ is equivalent to the cross-entropy loss. A different κ leads to a different feature distribution of the samples.

In general, this stage completes the joint supervised learning of the feature extractor and classifier on the labeled source samples and fixes the parameters of the modules, obtained by training, for the subsequent stages of the proposed method.

### 3.2. Joint Adversarial Domain Adaptation

The goal of this stage is to make sure the target feature extractor is set to minimize the distance of the marginal and conditional distributions between the source and target domains under their respective mappings, while maintaining the category discriminativeness to some extent in the target domain.

The details of this stage are shown in Figure 1. First, the parameters of the source feature extractor $\theta_{f}^{s}$ are used in this stage to initialize the target feature extractor $\theta_{f}^{t}$, because the target samples have no available labels. This may cause the gradient disappearance of the target feature extractor in the joint adversarial process, and thus, a degenerate solution may be learned. The domain discriminator $\theta_{d}$ is composed of three fully connected layers, and takes the feature representations $f^{s}$ and $f^{t}$ as the inputs, as shown in Figure 1. Because predicting the domain label is a two-class classification problem, the sigmoid function is used to map $\theta_{d}\left(f\right)$ between (0,1). Then, the probability of domain samples belonging to a particular domain discriminator is obtained, and the specific calculation is as follows:

(5) $$p\left({\hat{y}}_{d}\right)=\frac{1}{1+\exp\left(-\theta_{d}\left(f\right)\right)}$$

Second, the target feature extractor is also used to extract the features of the source samples and predict their specific category ${\hat{y}}_{c}^{s}$ to supervise the category-wise separability of the extracted features. We set up the confusion optimization goals of the target feature extractor and domain discriminator separately to align the marginal and conditional distributions simultaneously.

In the training process, the domain discriminator $\theta_{d}$ is optimized to minimize the domain classification loss, whereas the feature extractor $\theta_{f}^{t}$ is optimized to minimize the label prediction loss of the source samples and maximize the domain classification loss. We perform joint adversarial adaptation by learning $\theta_{f}^{t}$ such that the domain discriminator that sees the encoded source and target examples cannot reliably predict their domain label. Hyperparameter λ controls the trade-off between the two objectives that shape the features during the learning. The overall objective of the joint adversarial network is described as follows:

(6) $$\begin{array}{cc} L^{f} & =L_{adv}^{f}+\lambda L_{cls}^{f}\\ & =-\log(p({\hat{y}}_{d}^{t}))-\lambda \sum_{i=1}^{m}y_{i}^{s}\log{\hat{y}}_{i}^{s} \end{array}$$

(7) $$L_{adv}^{d}=-\log\left(p\left({\hat{y}}_{d}^{s}\right)\right)-\log(1-p({\hat{y}}_{d}^{t}))$$

where $L_{\mathrm{adv}\;}$ is the loss for the domain classification and $L_{cls}$ is the loss for label prediction. The joint adversarial network searches for $\theta_{f}^{t}$ and $\theta_{d}$ which generates a saddle point of $L^{f}$ and $L_{\mathrm{adv}\;}^{d}$ during the learning process, which can be described as follows:

(8) $$\theta_{f}^{t}=\underset{\theta_{f}^{t}}{\arg\min}L^{f}$$

(9) $$\theta_{d}=\underset{\theta_{d}}{\arg\min}L_{\mathrm{adv}\;}^{d}$$

Based on the above Equations (6) and (7), training is performed using the stochastic gradient descent (SGD) algorithm and the saddle point (8) and (9) can be found via updating as follows:

(10) $$\theta_{f}^{t}\leftarrow \theta_{f}^{t}-\eta \left(\frac{\partial L_{adv}^{f}}{\partial \theta_{f}^{t}}+\lambda \frac{\partial L_{cls}^{f}}{\partial \theta_{f}^{t}}\right)$$

(11) $$\theta_{d}\leftarrow \theta_{d}-\eta \left(\frac{\partial L_{adv}^{d}}{\partial \theta_{d}}\right)$$

where η represents the learning rate, which can vary over iterations.

Reviewing the whole process of the JADA stage, it can be found that no labeled samples in the target domain participate in the network training. The feature extractors $\theta_{f}^{s}$ and $\theta_{f}^{t}$ have the same network structure, but they do not share weights. For many previous joint adversarial adaptation methods [24], all layers are constrained, thus enforcing the exact source and target mapping consistency. However, this may make the optimization poorly conditioned, since the same network must handle samples from two separate domains. The proposed method has favored untying weights between the two domains, allowing models to learn parameters for each domain individually. Furthermore, it adapts both the marginal and conditional distributions between the source and target domains, and finally learns more separable domain-invariant features. In the following section, we diagnose the fault instances in the target domain.

### 3.3. Fault Identification

When diagnosing samples in the target domain, we first fix the parameters of the target feature extractor $\theta_{f}^{t}$ and classifier $\theta_{c}$ that were trained, and then input the time–frequency images $x^{t}$ of the target samples into the target feature extractor $\theta_{f}^{t}$ to obtain its feature representation $f^{t}$. Finally, we use the classifier to predict its category ${\hat{y}}_{c}^{t}$. This part of the calculation process is shown in Equation (12).

(12) $$\begin{array}{cc} {\hat{y}}_{c}^{t}=\underset{i}{\arg\max} & \left(\frac{\exp\left(\theta_{c}{\left(\theta_{f}^{t}\left(x^{t}\right)\right)}_{i}\right)}{\sum_{j=1}^{m}\exp\left(\theta_{c}{\left(\theta_{f}^{t}\left(x^{t}\right)\right)}_{j}\right)}\right),\\ & i=1,\ldots ,m \end{array}$$

## 4. Experiment and Result Analysis

In this section, we evaluate the efficacy of the JADA method on the benchmark rolling bearing dataset obtained from the Case Western Reserve University (CWRU) [25] and the unpublished rolling bearings dataset collected from the Drivetrain Diagnostics Simulator (DDS). We also perform an extensive empirical evaluation of the proposed approach with several popular DA methods.

### 4.1. Experiments on DDS Dataset

#### 4.1.1. Data Description

**Figure 3:**
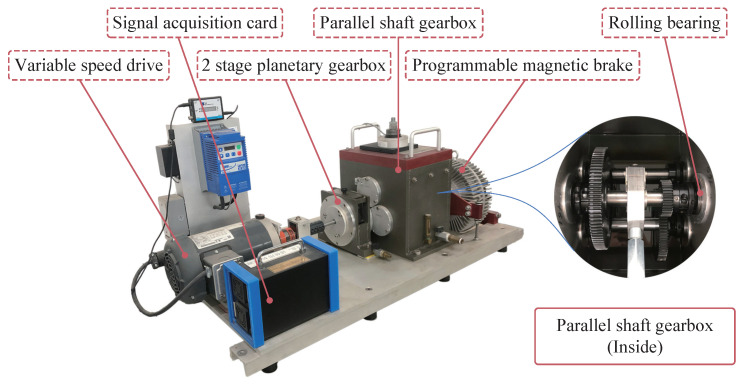
Drivetrain diagnostics simulator.

The dataset was collected from the DDS designed by Spectra Quest, as shown in Figure 3. This drivetrain consists of a two-stage planetary gearbox, two-stage parallel shaft gearbox with rolling bearings, bearing loader, and programmable magnetic brake.

**Figure 4:**
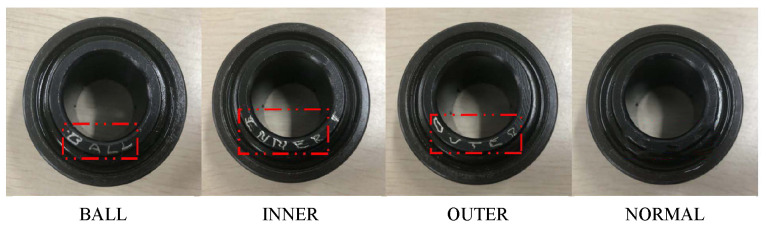
Four bearing health conditions.

Based on this drivetrain, we constructed four bearing health conditions by replacing the rolling bearings in the gearbox to simulate the industrial transmission system, as shown in Figure 4, including health (normal), inner race damage (inner), ball damage (ball), and outer race damage (outer). We applied a torsional load by controlling the 3HP variable frequency AC drive, and the experiments were carried out under 0, 4, 6, and 8 V.

The vibration data were acquired by using SQI608A11-3F unidirectional acceleration sensors which were mounted on both ends of the fixed shaft of the gearbox through bolt connection under different working conditions and at a sampling frequency of 20 kHz. The samples drawn from four different working conditions are: A, B, C, and D, as listed in Table 1. There were four categories under each domain, and each category had 410,624 data points. We applied a sliding window with a length of 2048 and 50% overlapping for the pre-processing, and 400 samples were assigned in each category.

As one of the frequently used time–frequency analysis techniques, short-time Fourier transform (STFT) was applied to all the samples to obtain the corresponding time-varying frequency spectrum information. The Hamming window was used as the window function, the length of the window function was pre-set to 120, and the window overlap was 50%. After converting the time-domain raw vibration signals into time–frequency images by STFT, we acquired images with a size of 64 × 64 × 3, which were input into the feature extractor to train the model.

#### 4.1.2. Transfer Diagnosis Tasks Settings

Because different operating conditions lead to an inconsistent distribution of the vibration data, twelve transfer diagnosis tasks under different scenarios can be constructed by the DDS dataset as listed in Table 2, e.g., $T_{BA}$ denotes that B is the source domain and A is the target domain. In any transfer diagnosis task, the training dataset comprises every labeled sample from the source domain and 75% of the unlabeled samples from the target domain, while the remaining unlabeled samples from the target domain are utilized for testing.

#### 4.1.3. Parameters of the Proposed Method

To achieve the best possible result, the parameters and implementation details of the JADA method are mainly determined based on the experiment results and relevant literature. The network is built according to the JADA fault diagnosis model structure described in Section 3, and the detailed architecture of JADA is listed in Table 3, which divides the model into four modules according to the functions of each part of the model, i.e., the source feature extractor, target feature extractor, classifier, and discriminator. The source and target feature extractors share the same architecture, which consists of two convolutional layers, two max-pooling layers and two fully connected layers. The input of the feature extractor is time–frequency images as mentioned before, and the output is a feature vector with a size 1 × 128. In addition, both the classifier and discriminator are composed of fully connected layers, and both take the feature vector, output by the feature extractor, as the input.

To improve the efficiency of model optimization, the hyperparameters are set as elaborated below based on the results of multiple experiments.

(1) Classifier pre-training stage: The Adam algorithm is selected as the optimizer, which dynamically adjusts the learning rate via first-order and second-order moment estimations. The initial learning rate is 0.0001, whereas the exponential decay rates of the first-order and second-order moment estimations are 0.9 and 0.999, respectively. Scalar α is selected by searching {0,0.01,0.05,0.1,0.5,1} and fixed as α=0.5.

(2) Joint adversarial adaptation stage: The Adam algorithm is selected to optimize the parameters of the target feature extractor and domain discriminator, where the initial learning rates of the target feature extractor and domain discriminator are 0.0001 and 0.0005, respectively. The exponential decay rates of the first-order and second-order moment estimations are set to 0.9 and 0.999, respectively.

In addition, the batch size is set as 64 for both the above-mentioned stages, whereas the classifier pre-training stage and joint adversarial adaptation stage trained 200 and 1000 iterations, respectively.

The hyperparameter κ in Equation (3) dominates the intra-class variations, and λ in Equation (8) is a trade-off parameter to balance the discrepancy between the marginal distribution and conditional distribution across the domains. Because both of them seriously affect the transfer performance of the JADA, we conducted two experiments to investigate their sensitivities.

**Figure 5:**
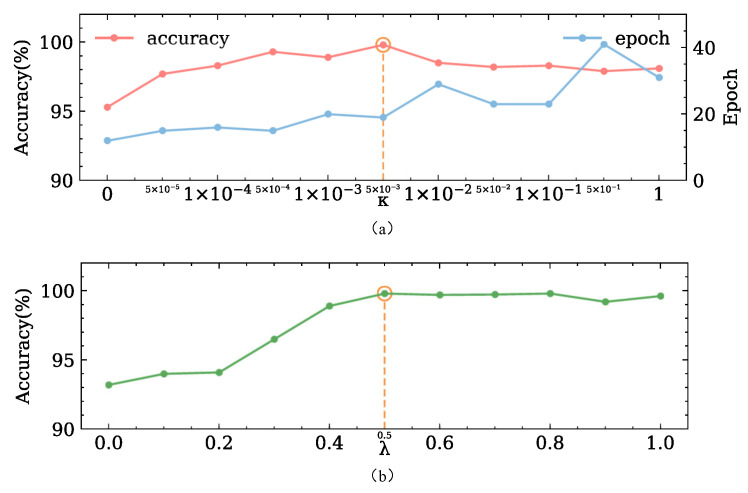
Diagnosis accuracies for the DDS dataset, respectively, achieved by (**a**) models with different κ and fixed λ=0.5. (**b**) models with different λ and fixed $K=5\times 10^{-3}$.

In the first experiment, we fixed λ=0.5 and varied κ to evaluate the performance of the learned models. The average classification accuracies of these models on twelve transfer diagnosis tasks are shown in Figure 5. It is obvious that simply using the cross-entropy loss (in this case, κ=0) results in a poor transfer performance. Properly choosing the value of κ can improve the classification accuracies of the JADA. We can observe that the model reaches its peak accuracy when κ is set to $5\times 10^{-3}$.

In the second experiment, we fixed $K=5\times 10^{-3}$ and varied λ from 0 to 1 to evaluate the performance of the learned models. It is obvious that only adapting the marginal distribution (in this case, λ=0) results in poor classification accuracy, which indicates that the class-wise distribution of the learned features is under-adapted. On the contrary, the model reaches its peak accuracy when λ is set to 0.5. Moreover, the transfer performance of JADA remains largely stable across a wide range of λ, which indicates that λ can balance the contributions of the marginal distribution and conditional distribution adaptations in the loss function.

To achieve the best transfer performance of the JADA, we set κ and λ to $5\times 10^{-3}$ and 0.5, respectively, based on the aforementioned analysis.

#### 4.1.4. Comparison Methods

To verify the effectiveness of the proposed method, we compared the classification accuracy and transfer performance of the proposed method with those of the other methods, including CNN, Transfer Component Analysis (TCA) [26], Joint Distribution Adaptation (JDA) [27], Domain Adversarial Neural Network (DANN) [28], and Adversarial Discriminative Domain Adaptation (ADDA) [29]:

(1) CNN: As a benchmark for evaluating the domain-invariant feature learning capabilities of the DA methods, CNN is trained on only the source samples, and then, the trained model is directly applied to the target data. The architecture of the CNN is the same as the backbone of JADA.

(2) TCA: TCA maps the source and target samples into reproducing a kernel Hilbert space using the kernel function to minimize the difference in marginal distribution between the source and target domains while retaining their internal attributes. The optimal subspace dimension is set by searching 4, 8, 16, 32, 64, 128, and the trade-off parameter is searched from 0.01, 0.1, 1, 10, 100, while using the linear kernel [30].

(3) JDA: JDA can adapt the marginal distribution and conditional distribution between the source and target domains simultaneously, and its hyperparameters are consistent with those of the TCA.

(4) DANN: DANN first leverages the adversarial learning between the domain discriminator and feature extractor to achieve domain-invariant representations, while the gradient reversal layer is introduced to automatically reverse the gradient direction of the domain classification loss during the back propagation process. The backbone architecture of the DANN is the same as that of the proposed method.

(5) ADDA: Tzeng et al. [28] summarized a general adversarial adaptation (GAN) framework, then proposed ADDA with a GAN-based loss, which learns the feature extractor through adversarial training and realizes the classification of the target samples by sharing the classifier.

For a fair comparison, the hyperparameters of all the aforementioned methods are determined based on experiments and reported literature to obtain the best classification accuracy for each transfer diagnosis task. Every experiment is repeated ten times to report the results for reducing the randomness and singularity. In addition, the network optimization part of the above-mentioned methods uses the Adam algorithm as the optimizer with a set learning rate of 0.0001.

#### 4.1.5. Result Analysis

**Figure 6:**
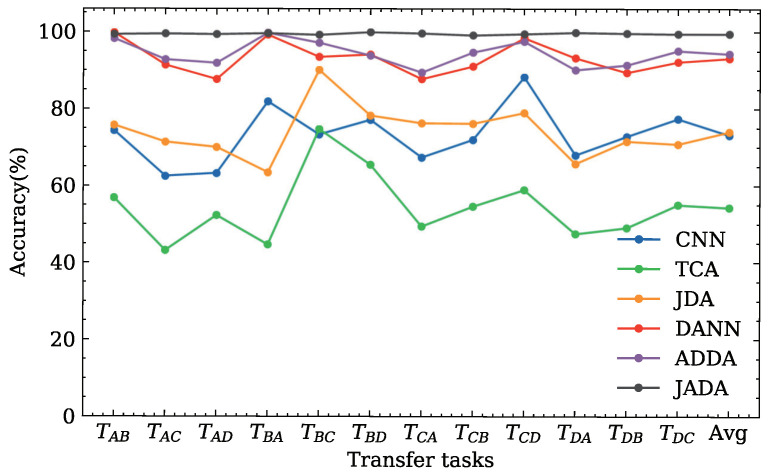
Classification accuracies of the different methods for the DDS dataset.

The classification accuracies for twelve transfer diagnosis tasks derived the DDS dataset are illustrated in Figure 6 and Table 4.

As evident from the result of the experiment shown in Figure 6 and Table 4, the performance of the CNN is poor in every transfer diagnosis task. This indicates that changing the working loads produces a certain effect on the data distribution between the source and target domains.

The traditional transfer learning methods, i.e., TCA and JDA, have poor performance in each transfer diagnosis task with average accuracies of approximately 54.33% and 74.05%, respectively. This indicates that the traditional transfer learning methods may be unable to extract the high-level features from the samples and may be unsuitable for dealing with complex transfer diagnosis tasks owing to the lack of a corresponding domain adaptation layer and only considering the probability distribution between the source and target domains.

The adversarial domain adaptation-based methods are superior to the CNN, TCA, and JDA, indicating that the adversarial domain adaptation is significant for practical diagnostic requirements. Among the three adversarial domain adaptation methods, i.e., DANN, ADDA, and JADA, it can be seen that the proposed method achieves the best classification performance according to the average classification accuracy. Although the other comparison methods obtain a higher accuracy compared to the proposed method in several tasks, e.g., ADDA achieves 99.73% in the transfer diagnosis task $T_{BA}$, there are large differences in different tasks for these methods. In contrast, JADA can obtain robust results in various transfer diagnosis tasks.

In summary, the proposed method can effectively deal with the transfer diagnosis tasks under varying working conditions.

**Figure 7:**
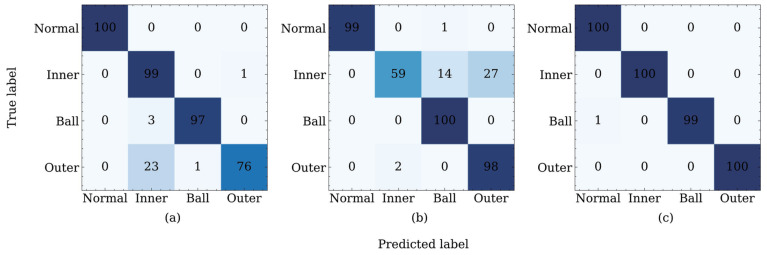
Confusion matrices of different methods (**a**) DANN, (**b**) ADDA, and (**c**) JADA.

For a detailed analysis of the classification accuracy of each category, we take the transfer diagnosis task $T_{DA}$ as an example and calculate the confusion matrix corresponding to adversarial domain adaptation methods with a higher average classification accuracy, as shown in Figure 7.

Figure 7a shows that in addition to the normal category, DANN exhibits different degrees of misclassifications for the other three categories. Among them, the error classification of the outer race damage is the most serious. Twenty-three samples are misclassified as inner race damages and one sample is misclassified as ball damages. The classification accuracy of the ADDA for the outer race damage is higher than that of the DANN, as shown in Figure 7b. The ADDA method incorrectly categorizes the two samples as inner race damages. However, the ADDA method exhibits a large error when classifying the inner race damages, as shown in Figure 7b. Only fifty-nine samples are correctly classified, among the total one hundred samples. Consequently, according to the confusion matrix shown in Figure 7c, the proposed JADA method can correctly classify the categories of normal, inner, and outer. Furthermore, there is only one misclassification in the sample, whose category is ball. In general, the classification accuracy of the JADA method in each category is close to or reaches 100%, and the number of misclassification samples is far lower than those in the DANN and ADDA methods; this result verifies the superiority of the JADA over these other methods.

**Figure 8:**
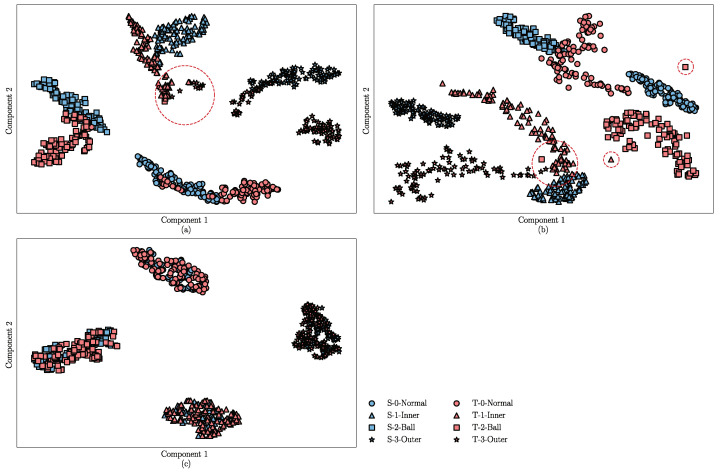
Feature visualization of the different methods for the DDS dataset: (**a**) DANN, (**b**) ADDA, and (**c**) JADA.

For a visual analysis of the DA and fault diagnosis performance of the DANN, ADDA, and the proposed method, the t-distributed stochastic neighbor embedding (t-SNE) algorithm [31] is introduced to reduce the dimension of the learned features and plot their distribution into a two-dimensional space according to the similarity. In this part, the feature extractor of the trained DANN, ADDA, and JADA methods are fixed, and then the target samples are used as the inputs. The learned features are shown in Figure 8a–c, where blue represents the source samples and red represents the target samples. Four different shapes are used to distinguish between the different categories of the samples.

The results shown in Figure 8a indicate that the features learned by the DANN exhibit good distinguishability in the source samples, however, there is a certain difference in the distribution of the target and source domains. Moreover, the features in the target domain are not well separated, and there are a few misclassifications, as shown in the red dashed circle in Figure 8a. The visualization results of the ADDA are shown in Figure 8b, where the boundary between the source domain features is clear, but there are several confusion and misclassifications in the target domain, as shown in the red dashed circle in Figure 8b. In addition, there is a huge discrepancy in the feature distribution between the source and target domains, possibly because the ADDA method ignores the discrepancy in the conditional distribution between the source and target samples. Figure 8c indicates that the learned transferable features are subject to smaller distribution discrepancies compared to those shown in Figure 8a,b, and the features of the source and target domains from the same category are densely clustered, which indicates that the proposed JADA can correct the distribution discrepancy between the features that are learned from the different domains. The result visually proves that the JADA method has a better transfer performance compared to the other methods.

### 4.2. Experiments on the CWRU Dataset

#### 4.2.1. Data Descriptions

**Figure 9:**
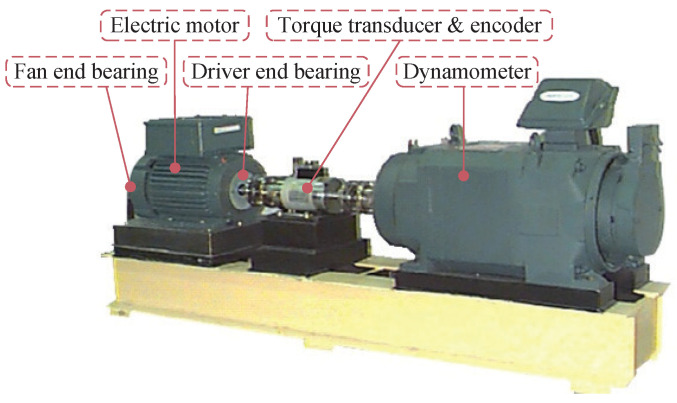
Experimental setup of motor bearing.

Considering that the open source dataset is of great significance for the evaluation and comparison of intelligent fault diagnosis methods, we selected the public rolling bearing dataset from CWRU as the second validation dataset. The vibration data of the CWRU dataset were collected using accelerometers, which were attached to the housing. As shown in Figure 9, the test stand consists of a motor, a torque transducer/encoder, a dynamometer, and control electronics.

The CWRU dataset is divided into normal data and faulty data. The fault data are generated by single-point damage at the inner raceway (IR), ball (B), and outer raceway (OR) of SKF6205 bearings. The single-point faults were introduced to the bearings using electro-discharge machining with fault diameters of 0.007, 0.014, and 0.021 in (1″ = 2.54 cm). In addition, the vibration data were recorded for motor loads of 0, 1, 2, and 3 horsepower (hp, 1 hp = 746 W), and the digital data were collected at 12,000 samples per second. According to different fault locations and fault diameters, we selected 10 types of data for experiments under four motor loads, as listed in Table 5; taking ‘IR007_1’ as an example, ‘IR’ denotes that the fault location of this category of fault is the inner raceway, ‘007’ indicates that the fault diameters of this fault is 0.007 in, and ‘_1’ indicates that the workload is 1 hp. Moreover, for each motor load, there are ten categories, and each category has 235 samples with a length 1024. During the experiment, these samples were subjected to STFT, and the specific transform setting were the same as those mentioned in Section 4.1.1. Furthermore, a total of 9400 time–frequency images in the CWRU dataset were obtained.

#### 4.2.2. Transfer Diagnosis Tasks Settings

In this part, twelve transfer diagnosis tasks under different scenarios can be constructed by the CWRU dataset, namely $T_{01}$, $T_{02}$, $T_{03}$, $T_{10}$, $T_{12}$, $T_{13}$, $T_{20}$, $T_{21}$, $T_{23}$, $T_{30}$, $T_{31}$, and $T_{32}$, where $T_{ij}$ denotes that all the samples under *i* hp are used as the source domain, and all samples under jhp are used as the target domain. The source data are labeled while the target domain data are unlabeled.

#### 4.2.3. Result Analysis

**Figure 10:**
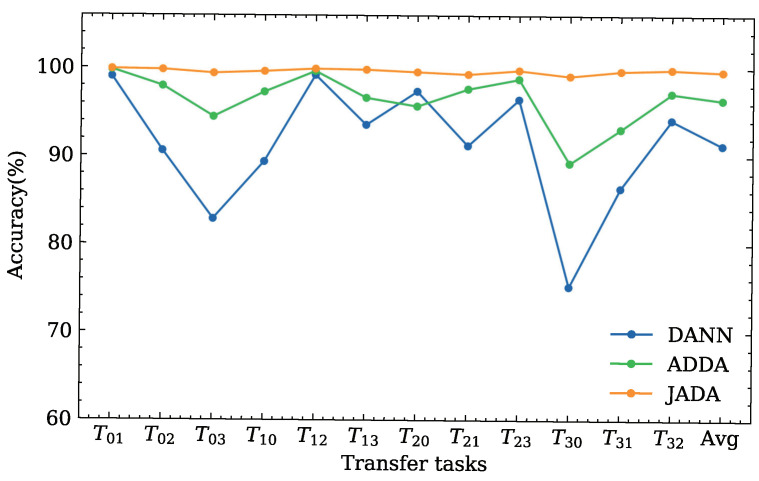
Classification accuracies of the different methods for the CWRU dataset.

In the above comparative experiments, the accuracy of the three adversarial domain adaptation methods, i.e., DANN, ADDA, and JADA, is significantly higher than other methods. Therefore, we only compare the classification accuracy and transfer performance of the proposed method with those of the adversarial DA methods for the twelve transfer diagnosis tasks of the CWRU dataset, as illustrated in Figure 10 and Table 6.

It can be seen that the average accuracy of the adversarial-based methods participating in the comparison is higher than 90%. Compared with twelve transfer tasks horizontally, the classification accuracy of the proposed method is better than that of the other two methods, and we can see that the proposed method is superior to the competing methods in most scenarios, as shown in Figure 10. The average accuracy of the proposed method is 99.67%, which is higher than those reported in [7,32] (99.2% and 99.3%). In these reported studies [7,31], the transfer diagnosis task settings were the same as those used in our experiment. This result further verifies the superiority of the proposed JADA method.

**Figure 11:**
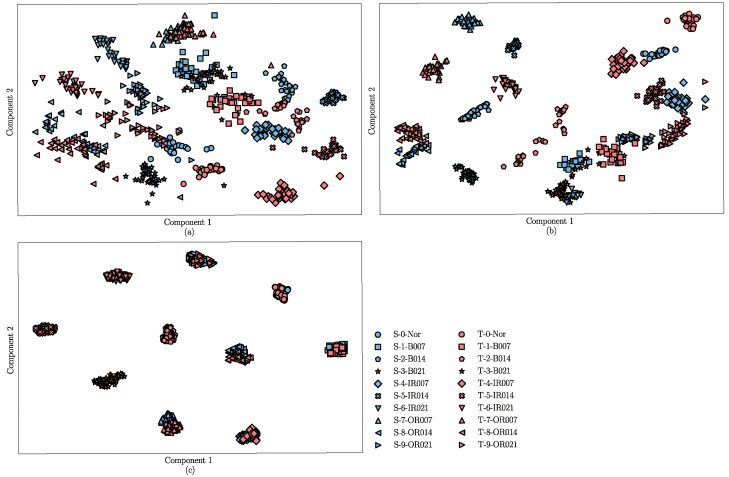
Feature visualization of different methods for the CWRU dataset: (**a**) DANN, (**b**) ADDA, and (**c**) JADA.

Furthermore, we take the transfer diagnosis task $T_{30}$ as an example, and visualize the learned features of the three methods using the t-SNE algorithm, as shown in Figure 11. In the figure, the source samples are represented by blue and the target samples are represented by red.

We can make intuitive observations: (1) Figure 11a shows that the learned features are mixed, implying that the DANN cannot discriminate both the source and target samples very well. (2) Figure 11b shows that the ADDA discriminate the source domain well, but the learned features of most target samples are away from the right source class and are even close to the wrong source classes. This reveals that the ADDA method cannot effectively align the marginal distribution and conditional distribution across the source and target domains. (3) Figure 11c demonstrates that the JADA can discriminate between different classes in both the source and target domains when the target samples are close to the right source classes. These results demonstrate the efficacy of joint adversarial adaptation and the category center constraint.

## 5. Conclusions

This paper presents a novel JADA method for cross-condition fault diagnosis. Unlike the previous adversarial adaptation methods that ignored the class-wise mismatch across domains and resulted in inaccurate distribution alignments, the proposed JADA method can align the marginal distribution and conditional distribution across the source and target domains simultaneously through a unified adversarial learning process and promotes positive transfer by minimizing the distance within each category in the shared feature space. The proposed method successfully achieves accurate classification results and a satisfactory domain adaptation ability.

## Figures and Tables

**Table 1:** Data description of the DDS dataset.

Fault Class	Domain			
	A(0V)	B(4V)	C(6V)	D(8V)
Normal	Normal_0	Normal_4	Normal_4	Normal_4
Inner race	Inner_0	Inner_4	Inner_4	Inner_4
Ball	Ball_0	Ball_4	Ball_4	Ball_4
Outer race	Outer_0	Outer_4	Outer_4	Outer_4

**Table 2:** Description of the transfer diagnosis tasks for the DDS dataset.

Transfer Tasks	$T_{\mathrm{AB}}$	$T_{\mathrm{AC}}$	$T_{\mathrm{AD}}$	$T_{\mathrm{BA}}$	$T_{\mathrm{BC}}$	$T_{\mathrm{BD}}$	$T_{\mathrm{CA}}$	$T_{\mathrm{CB}}$	$T_{\mathrm{CD}}$	$T_{\mathrm{DA}}$	$T_{\mathrm{DB}}$	$T_{\mathrm{DC}}$
Source domain	A	A	A	B	B	B	C	C	C	D	D	D
Target domain	B	C	D	A	C	D	A	B	D	A	B	C

**Table 3:** Hyperparameters of JADA.

Module	Layer Type	Activation Function	Kernel Size	Stride	Output Size
Feature extractor	Conv_1	relu	3 × 3	1	(64, 64, 16)
	Batch Norm	/	/	/	(64, 64, 16)
	Max-pooling	/	3 × 3	2	(32, 32, 16)
	Conv_2	relu	3 × 3	1	(32, 32, 64)
	Batch Norm	/	/	/	(32, 32, 64)
	Max-pooling	/	3 × 3	2	(16, 16, 64)
	Flatten	/	/	/	(1, 16 × 16 × 64)
	FC_1	relu	/	/	(1, 256)
	FC_2	tanh	/	/	(1, 128)
Classifier	FC_3	softmax	/	/	(1, 4)
Discriminator	FC_4	Leaky Relu	/	/	(1, 128)
	FC_5	Leaky Relu	/	/	(1, 128)
	FC_6	sigmoid	/	/	(1, 1)

**Table 4:** Classification accuracies of the different methods for transfer diagnosis tasks (%).

Method	$T_{\mathrm{AB}}$	$T_{\mathrm{AC}}$	$T_{\mathrm{AD}}$	$T_{\mathrm{BA}}$	$T_{\mathrm{BC}}$	$T_{\mathrm{BD}}$	$T_{\mathrm{CA}}$	$T_{\mathrm{CB}}$	$T_{\mathrm{CD}}$	$T_{\mathrm{DA}}$	$T_{\mathrm{DB}}$	$T_{\mathrm{DC}}$	Avg
CNN	74.32	62.45	63.19	81.87	73.29	77.11	67.32	71.93	88.26	67.95	72.80	77.41	73.16
TCA	56.87	43.20	52.31	44.70	74.73	65.47	49.43	54.62	58.93	47.52	49.10	55.06	54.33
JDA	75.76	71.38	70.02	63.48	90.10	78.25	76.28	76.16	78.97	65.76	71.59	70.85	74.05
DANN	99.76	91.38	87.66	99.23	93.48	94.11	87.74	91.03	98.42	93.23	89.45	92.21	93.14
ADDA	98.25	92.82	91.92	99.73	97.16	93.87	89.49	94.70	97.53	90.18	91.44	95.13	94.35
JADA	99.35	99.50	99.35	99.61	99.23	99.92	99.61	99.13	99.47	99.84	99.65	99.49	99.51

**Table 5:** Data description of the CWRU dataset.

Fault Locations	Motor Loads			
	0 hp	1 hp	2 hp	3 hp
Normal	Nor_0	Nor_1	Nor_2	Nor_3
IR	IR007_0	IR007_1	IR007_2	IR007_3
	IR014_0	IR014_1	IR014_2	IR014_3
	IR021_0	IR021_1	IR021_2	IR021_3
B	B007_0	B007_1	B007_2	B007_3
	B014_0	B014_1	B014_2	B014_3
	B021_0	B021_1	B021_2	B021_3
OR	OR007_0	OR007_1	OR007_2	OR007_3
	OR014_0	OR014_1	OR014_2	OR014_3
	OR021_0	OR021_1	OR021_2	OR021_3

**Table 6:** Diagnosis results for the cross-domain bearing datasets (%).

Transfer Diagnosis Tasks	DANN	ADDA	JADA
$T_{01}$	99.03	99.81	99.87
$T_{02}$	90.61	97.95	99.80
$T_{03}$	82.86	94.46	99.40
$T_{10}$	89.37	97.29	99.62
$T_{12}$	99.22	99.66	99.92
$T_{13}$	93.58	96.65	99.84
$T_{20}$	97.39	95.71	99.59
$T_{21}$	91.24	97.68	99.35
$T_{23}$	96.49	98.81	99.80
$T_{30}$	75.23	89.27	99.16
$T_{31}$	86.41	93.13	99.73
$T_{32}$	94.18	97.21	99.91
Avg	91.30	96.46	99.67

## Data Availability

This study did not report any data.
